# Phase IIa Clinical Trial of Trans-1-Amino-3-^18^F-Fluoro-Cyclobutane Carboxylic Acid in Metastatic Prostate Cancer

**Published:** 2014

**Authors:** Yusuke Inoue, Yuji Asano, Takefumi Satoh, Ken-ichi Tabata, Kei Kikuchi, Reiko Woodhams, Shiro Baba, Kazushige Hayakawa

**Affiliations:** 1 Department of Diagnostic Radiology, Kitasato University School of Medicine, Sagamihara, Kanagawa, Japan; 2 Department of Urology, Kitasato University School of Medicine, Sagamihara, Kanagawa, Japan; 3 Department of Radiology, Kitasato University Hospital, Sagamihara, Kanagawa, Japan; 4 Department of Radiation Oncology, Kitasato University School of Medicine, Sagamihara, Kanagawa, Japan

**Keywords:** Anti-^18^F-FACBC, Metastasis, Positron Emission Tomography, Prostate cancer

## Abstract

**Objective(s):**

We performed a phase IIa clinical trial of trans-1-amino-3-^18^F-fluoro-cyclobutane carboxylic acid (anti-^18^F-FACBC), a synthetic amino acid analog for PET, in patients with metastatic prostate cancer.

**Methods:**

The study subjects consisted of 10 untreated prostate cancer patients having lymph node and/or bone metastasis. Five patients underwent whole-body PET 5 and 30 min after intravenous injection of anti-^18^F-FACBC. The other five patients underwent 60 min dynamic PET of the pelvis. Safety assessment was performed before and 24 h after injection. PET/CT images were assessed visually, and time courses of anti-^18^F-FACBC uptake were evaluated from dynamic imaging.

**Results:**

Two mild adverse events were observed and resolved without treatment. All 10 patients showed increased accumulation of anti-^18^F-FACBC in the primary prostate lesion. CT revealed five enlarged lymph nodes indicating metastasis, and all showed increased uptake. Additionally, anti-^18^F-FACBC PET delineated unenlarged lymph nodes as hot spots. Anti-^18^F-FACBC PET demonstrated metastatic bone lesions, similar to conventional imaging. In one of two patients with lung metastasis, some lesions showed increased uptake. Regarding the time course, increased uptake of anti-^18^F-FACBC in the lesion was demonstrated immediately after injection, followed by gradual washout.

**Conclusion:**

The results of this phase IIa clinical trial indicated the safety of anti-^18^F-FACBC in patients with prostate cancer and the potential of anti-^18^F-FACBC PET to delineate primary prostate lesions and metastatic lesions. This clinical trial was registered as JapicCTI-101326.

## Introduction

Imaging modalities play an important role in the staging and restaging of prostate cancer (1), and use of positron emission tomography (PET) has been investigated (2, 3). Although 2-deoxy-2-[^18^F]fluoro-D-glucose is used in various malignant neoplasms, it is not useful in prostate cancer because of low tumor uptake and high urinary excretion. Better diagnostic performance is expected for ^11^C-acetate and ^11^C-choline; however, these ^11^C-labeled tracers require an in-house cyclotron, which has restricted their widespread use. Although ^18^F-fluorocholine is excreted rapidly in urine unlike ^11^C-choline, the longer half-life of ^18^F offers convenience, and its application to prostate cancer is gaining increasing interest (4).

The synthetic amino acid analog trans-1-amino-3-^18^F-fluoro-cyclobutane carboxylic acid (anti-^18^F-FACBC) is another candidate for PET imaging of prostate cancer. Rapid, high uptake of anti-^18^F-FACBC was shown in prostate cancer (5-7), and its usefulness for the assessment of recurrent prostate cancer has been demonstrated (8-11). The phase I clinical trial of anti-^18^F-FACBC (development code, NMK36) in Japan indicated safety, acceptable radiation dose, and favorable characteristics for imaging brain and pelvic tumors (12). As a next step, we performed a phase IIa clinical trial in patients with metastatic prostate cancer to evaluate the safety of anti-^18^F-FACBC in patients with metastatic prostate cancer and to examine the capability of anti-^18^F-FACBC PET to delineate primary and metastatic lesions of prostate cancer.

## Methods

### Investigational Drug

Anti-^18^F-FACBC was prepared by Nihon Medi-Physics Co., Ltd. (Tokyo, Japan) based on the method reported previously (13), and was supplied in a vial containing 2 ml solution with additives to ensure high stability for commercial distribution. The radiochemical purity was 99.5 ± 0.1%.

### Subjects

Ten male patients with prostate cancer aged 69.9 ± 7.9 years (mean ± SD) were enrolled in this study between November 2010 and March 2011 (Table 1). The inclusion criteria were: a) histological positivity for prostate cancer on two or more cores obtained by needle biopsy of the prostate; and b) presence of lymph node metastasis and/or bone metastasis demonstrated on conventional imaging such as computed tomography (CT), magnetic resonance (MR) imaging, and bone scintigraphy. Patients, who had undergone therapy for prostate cancer, including surgical resection, radiation therapy, hormonal therapy, and chemotherapy, were excluded. All patients underwent anti-^18^F-FACBC PET after an interval of more than 3 weeks after biopsy to avoid possible effects of biopsy on PET images. The study was performed after approval by the institutional review boards of the host institution and according to Good Clinical Practice (GCP Ordinance No. 28, 27, March 1997). Written informed consent was obtained from all patients before participation. This clinical trial was registered as JapicCTI-101326.

**Table 1:** Patient Characteristics

Patient. No.	Group	Age	Sex	BH (cm)	BW (kg)	Dose (MBq)	PSA (ng/ml)	Positive biopsy	Gleason score
**1**	WB	75	M	158.5	45.1	93.8	452.9	6/8	4+4
**2**	WB	65	M	166.7	57.8	130.2	2177.6	8/8	5+4
**3**	WB	65	M	157.8	73.6	164.7	647.0	5/8	4+5
**4**	WB	75	M	168.0	69.0	217.5	111.3	6/6	4+5
**5**	WB	54	M	159.7	69.9	287.8	13.5	7/8	5+4
**6**	Dyn	70	M	174.0	78.1	201.9	4.8	3/8	3+4
**7**	Dyn	75	M	164.8	66.0	194.8	134.5	4/8	4+3
**8**	Dyn	75	M	161.1	63.2	202.4	104.6	8/8	4+4
**9**	Dyn	81	M	149.0	57.6	194.3	41.1	9/10	5+5
**10**	Dyn	64	M	166.0	65.2	196.1	182.0	10/10	5+4

BH: body height, BW: body weight, WB: whole-body group, Dyn: dynamic group.

Positive biopsy indicates the number of cancer-positive biopsy cores and total cores obtained.

### PET/CT Procedures

Anti-^18^F-FACBC was injected after fasting for 4 h or longer, followed by PET/CT imaging using a TruePoint Biograph 6 PET/CT scanner (Siemens Medical Solutions, Hoffman Estates, IL). For CT, 5 mm thick contiguous slices were acquired without the administration of contrast medium. The initial five patients underwent whole-body PET imaging (whole-body group), and the injected dose ranged from 93.8 to 287.8 MBq. Whole-body PET imaging was performed 5 min (early) and 30 min (late) after injection. Data were acquired from the mid-thigh level to the top of the head for 2.5 min per bed position. The remaining five subjects underwent 60 min dynamic PET imaging of the pelvic region immediately after injection (dynamic group), and the injected dose was fixed at approximately 200 MBq (194.3–202.4 MBq). Images for visual evaluation were reconstructed using data of 2.5 min duration and centered at 5, 15, 30, and 58 min after injection. Additionally, images were reconstructed at 15 s/frame × 4, 30 s/frame × 4, 2 min/frame × 6, 3 min/frame × 5, and 5 min/frame × 6 to assess the time course quantitatively.

### Safety Assessment

The following assessments were performed before and 24 h after injection to determine the safety of a single injection of anti-^18^F-FACBC: interview of subjective symptoms, physical examinations, 12 lead electrocardiography, measurements of blood pressure and pulse rate, hematology, blood chemistry, and urinalysis. An interview of subjective symptoms and physical examinations were also performed at the completion of PET/CT imaging.

### Conventional Imaging

CT, MR imaging, and bone scintigraphy were performed within 1 month before PET/CT with anti-^18^F-FACBC. In CT, after contrast injection, contiguous thin slices of 1.25 mm thickness were acquired from the neck to the pelvis. In MR imaging, T1-weighted imaging, T2-weighted imaging, diffusion-weighted imaging, and dynamic contrast-enhanced T1-weighted imaging of the pelvis were performed.

Although primary lesions were histologically proven to be prostate cancer, histological evaluation of metastatic lesions was not performed and results of these conventional imaging were used as the diagnostic standard. A lymph node with a short-axis diameter ≥ 10 mm on CT was judged to be significantly enlarged and regarded as a metastatic lymph node (14). The evaluation of bone metastasis was based primarily on bone scintigrams, with reference to CT and MR images. Patients were classified into three categories: multiple bone metastases (>20 lesions), limited bone metastasis (≤20 lesions), and no bone metastasis. In patients with limited bone metastasis, the locations of metastatic bone lesions were recorded. The locations were not considered in the analysis of patients with multiple bone metastases.

Judgment was performed by two board-certified radiologists, referring to official reports described by other board-certified radiologists. For inconclusive judgments, a third board-certified radiologist made the final decision.

### Visual Interpretation of PET/CT images

PET/CT images with anti-^18^F-FACBC were evaluated visually by two board-certified radiologists experienced in nuclear medicine, and discrepancies were resolved by consensus between them. The presence or absence of increased accumulation in the primary prostate lesion was determined with reference to MR images. Increased accumulation in the lymph node was also assessed on PET/CT images, and the results were compared with judgments of lymph node metastasis on CT. A focal area of increased anti-^18^F-FACBC accumulation in the bone was regarded as bone metastasis. Similar to judgments using conventional imaging methods, the locations of metastatic bone lesions were analyzed in detail only in patients with less than 20 metastatic bone lesions. Metastasis to other organs was also evaluated.

### Time Courses of Anti-^18^F-FACBC Uptake

Time courses of standardized uptake values (SUVs) were determined from dynamic PET imaging in five dynamic group patients. Spherical volumes of interest (VOIs) with approximately 1 cm diameters were placed in the primary prostate lesions, metastatic lymph nodes, and metastatic bone lesions, including an area of maximal activity for each lesion, and the maximum SUV (SUV_max_) was obtained. Activity excreted in the bladder was identified visually, and a 1 cm spherical VOI was set to include an area representing the earliest appearance in the bladder. A 1 cm spherical VOI was placed in the bone marrow of each side of the iliac bones, and a 2 cm spherical VOI was placed in each side of the gluteal muscles. The mean SUV (SUV_mean_) was calculated to represent activity in the bladder, bone marrow, or muscle. Mean values computed from two VOIs of each patient were used for the bone marrow and muscle.

## Results

### Safety Assessment

Two of the 10 patients experienced mild adverse events after anti-^18^F-FACBC injection. One patient complained of drowsiness at the completion of PET/CT imaging, which was resolved spontaneously on the following day. Lack of sleep due to pain from bone metastasis was thought to be the cause of the drowsiness. In the other patient, occult blood in urine was noticed on the day after anti-^18^F-FACBC injection, and disappeared 3 days later without treatment. A minor urinary tract infection was suspected in this patient based on slight increases in white blood cells and bacteria in urine, and was thought to be the cause of the urinary occult blood. These two events were judged to be clinically insignificant and to have no causal relationship with anti-^18^F-FACBC injection.

### Primary Lesion

**Figure 1:**
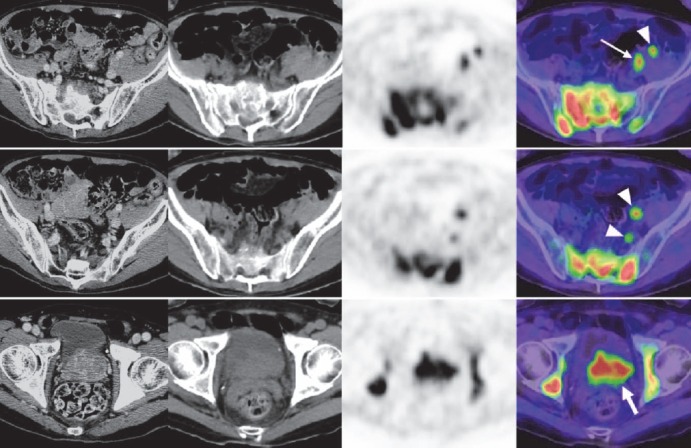
Axial images of the pelvis in a patient with lymph node metastasis and multiple bone metastases (patient no. 2 in Table 1). From left to right, contrast-enhanced thin-slice CT images, plain CT images of anti-^18^F-FACBC PET/CT, PET images, and PET/CT fusion images are presented. Images at a given body level are presented in a row. Contrast-enhanced CT and PET/CT were performed on different occasions. The primary site (thick arrow), a significantly enlarged lymph node in the left external iliac region (thin arrow), unenlarged lymph nodes in the left external and internal iliac regions (arrowheads), and multiple bone lesions showed increased uptake

**Figure 2:**
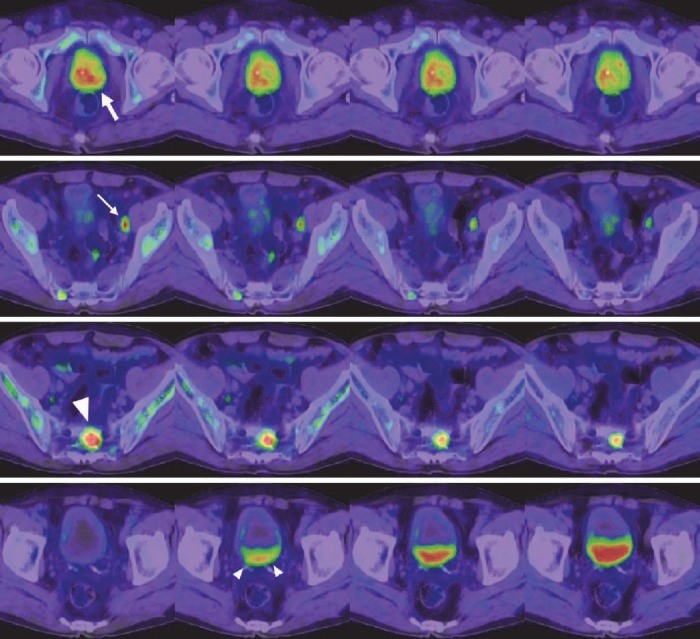
Axial fusion images of anti-^18^F-FACBC PET/CT in a patient with lymph node and limited bone metastasis (patient no. 10 in Table 1). From left to right, 5 min, 15 min, 30 min, and 58 min images are presented. On the 5 min images, the primary site (thick arrow), significantly enlarged lymph node in the left external iliac region (thin arrow), and sacral bone lesion (large arrowhead) showed increased uptake. Activity decreased over time; however, it was still evident on the 58-min images. Urinary excretion in the bladder is noted on the 15 min image (small arrowheads), followed by a gradual increase

All 10 patients showed increased accumulation of anti-^18^F-FACBC in the primary prostate lesion (Figure 1, 2). In the five patients of the whole-body group, increased accumulation was demonstrated on both early and late images, irrespective of injected dose. High radioactivity in the bladder, indicating urinary excretion, was identified in two and five patients on early and late images, respectively. Increased accumulation in the primary lesion was also shown in the five dynamic group patients, except on 58 min images in one patient. Bladder activity was not noted on 5 min images but was observed on 15 min images in all the five patients (Figure 2).

### Lymph Node Metastasis

CT demonstrated two enlarged lymph nodes in two patients of the whole-body group and three enlarged lymph nodes in three patients of the dynamic group. In total, five enlarged lymph nodes were detected and judged as metastasis. They were all located in the pelvis and included in the field of view (FOV) of PET. Their short-axis diameters were 10, 12, 12, 13, and 30 mm. One patient in the dynamic group was enrolled in the study based on enlargement of a lymph node; however, the short-axis diameter of the node was measured to be <10 mm on the final assessment. As a result, the patient was judged to have no metastatic lesion.

Increased uptake of anti-^18^F-FACBC was observed in the five metastatic lymph nodes (Figure 1, 2). Increased uptake was shown on both early and late images in the whole-body group and at all phases in the dynamic group. Additionally, increased uptake was detected in 14 unenlarged lymph nodes of eight patients with short-axis diameters of 4–9 mm (Figure 1). These lymph nodes were all located in the pelvic cavity. They were visualized during the early period, and some disappeared subsequently. In the whole-body group, 10 hot, unenlarged lymph nodes were shown in five patients on early images and eight of them remained hot on late images. In the dynamic group, four hot, unenlarged lymph notes were detected in three patients on 5 and 15 min images, and three and two of the four nodes exhibited increased uptake on 30 and 58 min images, respectively.

### Bone Metastasis

Conventional imaging in the whole-body group revealed multiple bone metastases (>20 sites) in four patients and limited bone metastasis in the other patient. In the dynamic group, no metastatic bone lesion was found in three patients, and limited bone metastasis was shown in two patients. One of these two patients had a solitary lesion in the left pubic bone that was located in the FOV of PET/CT. In the other patient, conventional imaging demonstrated sacral metastasis in the FOV of PET/CT, as well as metastatic lesions outside the FOV of PET/CT.

**Figure 3:**
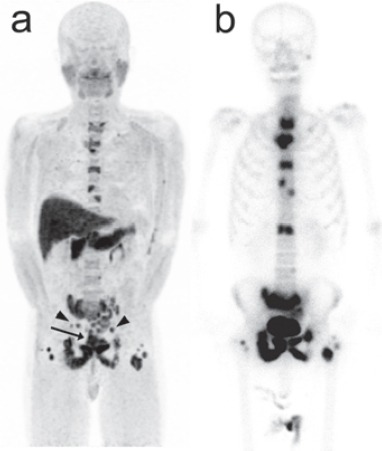
Anterior maximum-intensity projection image of anti-^18^F-FACBC PET (a) and anterior bone scintigram (b) in the patient presented in Figure 1. PET shows multiple areas of increased bone uptake, similar to bone scintigraphy. Increased uptake is also demonstrated in the primary site (arrow) and lymph nodes (arrowheads). Intense physiological uptake of anti-^18^F-FACBC is noted in the pancreas and liver

In four patients in whom conventional imaging revealed multiple bone metastases, anti-^18^F-FACBC PET demonstrated multiple sites of increased uptake indicating multiple bone metastases (Figure 3). In three patients, neither conventional imaging nor PET showed bone metastasis. In the remaining three patients, both conventional imaging and PET revealed limited bone metastasis (Figure 2). The locations of bone metastasis were concordant between conventional imaging and PET as far as the metastatic sites were within the FOV of PET. Judgments were consistent irrespective of the timing of PET imaging after injection.

### Lung Metastasis

**Figure 4:**
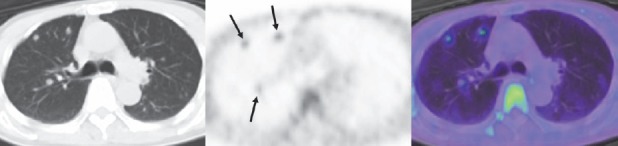
Axial images of the chest in the patient presented in Figure 1. From left to right, a CT image of anti-^18^F-FACBC PET/CT, PET image, and PET/CT fusion image are presented. Increased uptake is shown in tiny lung nodules (arrows). The same scale was used for presentation of PET in this figure as in Figure 1

Two patients in the whole-body group showed multiple lung metastases on CT. In one of the two patients (Figure 4), anti-^18^F-FACBC PET demonstrated some of the lesions, on the early and late images similarly. The long-axis diameters of lesions visualized on PET ranged from 4 to 12 mm. The long-axis diameter of the maximal invisible lung metastasis was 10 mm in the patient with PET visible lesions and 5 mm in the other patient. No increased activity indicating metastasis to other organs was observed.

### Time Courses of Anti-^18^F-FACBC Uptake

**Figure 5:**
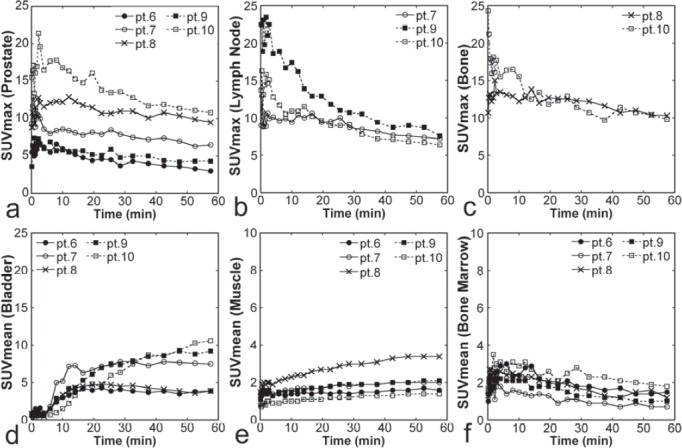
Time courses of SUVs from dynamic Imaging. SUV_max_ for primary prostate lesions (**a**, n = 5), SUV_max_ for metastatic lymph nodes (**b**, n = 3), SUV_max_ for metastatic bone lesions (**c**, n = 2), SUV_mean_ for the bladder (**d**, n = 5), SUV_mean_ for the muscle (**e**, n = 5), SUV_mean_ for the bone marrow (**f**, n = 5) are presented for each patient. The patient number (for example, pt. 6) corresponds to that in Table 1. Note that the scales of panels E and F are different from those of panels a-d

Increased uptake of anti-^18^F-FACBC was shown for primary prostate lesions, metastatic lymph nodes, and metastatic bone lesions immediately after injection, followed by gradual washout (Figure 2, 5). A relatively rapid washout was noted in two of the three metastatic lymph nodes. Urinary activity appeared in the bladder at about 5 min. Uptake in the gluteal muscles was low initially but increased slightly over time. Bone marrow uptake was above the background level initially and peaked at about 4 min, followed by a gradual decrease.

## Discussion

The phase I clinical trial of anti-^18^F-FACBC in Japan indicated its safety in six young adults (12). In the present phase IIa clinical trial, safety was tested in 10 patients with metastatic prostate cancer. Mild adverse events were noted in two patients, however, they resolved spontaneously without treatment, suggesting the safety of a single intravenous injection of anti-^18^F-FACBC in patients with prostate cancer. PET images of diagnostic quality were acquired after injection of anti-^18^F-FACBC ranging from 93.8 to 287.8 MBq in dose. Avidity of anti-^18^F-FACBC to primary prostate lesions, metastatic lymph nodes, and metastatic bone lesions was indicated. The study population was small, predominantly included patients with high Gleason scores, and did not include control subjects. Despite these limitations, the results of the present study, in combination with those of previous studies (8-11), warrant further investigation of diagnostic performance of anti-^18^F-FACBC PET in comparison with conventional imaging.

Anti-^18^F-FACBC accumulated in primary prostate lesions in all 10 patients during the early period. Because increased uptake areas were not correlated with histologically proven cancer tissues regarding localization within the prostate, the increased uptake may have partly represented accumulation in benign tissues. Accumulation of anti-^18^F-FACBC has been shown to be increased in benign prostatic hyperplasia as well as prostate cancer (7), and overlap of the intensity of accumulation has been demonstrated between malignant and non-malignant prostate tissues (15). Although anti-^18^F-FACBC PET is not specific for prostate cancer, it might play a role complementary to MR imaging in localizing cancer foci within the prostate (7) or might be used to guide biopsy to the most aggressive lesion (15).

On CT, significantly enlarged lymph nodes are considered to be malignant using a threshold short-axis diameter of 1 cm (14). Thus, small metastatic lesions are inevitably missed. In the present study, all five lymph nodes judged to be metastatic on CT showed increased uptake of anti-^18^F-FACBC. Previous studies have demonstrated the usefulness of anti-^18^F-FACBC PET in the detection of lymph node metastasis from prostate cancer (10, 11) and our observations are consistent with the previous reports. Additionally, 14 unenlarged lymph nodes were delineated as increased uptake areas. Because of the lack of histological confirmation, we cannot determine whether these increased uptakes represented true-positive or false-positive findings. However, these observations suggest the potential of anti-^18^F-FACBC PET to detect small lymph node metastasis and warrant further investigation to define its performance for the evaluation of nodal status. Although intestinal uptake was frequent, it was mild and did not interfere with the recognition of nodal uptake. In addition, rapid blood clearance appears to have aided the detection of accumulation in small lymph nodes.

A greater detection rate of bone lesions in prostate cancer recurrence has been reported using anti-^18^F-FACBC than using ^11^C-choline (10). In the present trial, classification of skeletal status into three categories, multiple bone metastases, limited bone metastasis, and no bone metastasis, was concordant between conventional imaging and anti-^18^F-FACBC PET. The locations of bone metastasis were also concordant in patients with limited bone metastasis as far as the lesions were located within the FOV of PET. These results support avidity of anti-^18^F-FACBC to metastatic bone lesions. Metastatic bone lesions from prostate cancer can be detected effectively by bone scintigraphy because they induce strong osteoblastic activity and consequent intense uptake of a bone-seeking agent. Sensitivity to bone metastasis should be compared between anti-^18^F-FACBC PET and bone scintigraphy in the future. Bone scintigraphy reflects bone reaction caused by a bone tumor, but does not directly visualize the bone tumor. PET with a tumor-seeking agent including anti-^18^F-FACBC is expected, at least, to play complementary roles in the differentiation of benign and malignant bone lesions and the evaluation of viability after therapy.

Increased uptake of anti-^18^F-FACBC in visceral metastases has not been described in the literature. In the present study, anti-^18^F-FACBC PET delineated some of tiny lung metastases and further support the avidity of anti-^18^F-FACBC PET to prostate cancer tissues. In detecting lung metastasis, CT is highly sensitive and the role of anti-^18^F-FACBC PET would be limited. However, anti-^18^F-FACBC PET may provide a clue to the detection and aid the differentiation of benign and metastatic lung nodules.

Previous studies demonstrated that accumulation of anti-^18^F-FACBC in prostate cancer lesions peaked within 5 min (5-7). In the present trial, high lesional uptake was observed immediately after injection, suggesting no need for a waiting time, and high activity in the lesions lasted for a long period. Whereas early imaging would be better to detect strong lesional activity and to avoid disturbance of lesion recognition by urinary excretion, the imaging time window may not be strictly restricted to an early period.

## Conclusion

The present phase IIa clinical trial indicated the safety of intravenous anti-^18^F-FACBC injection in patients with metastatic prostate cancer and the capability of anti-^18^F-FACBC PET to delineate primary prostate lesions and metastatic lesions including those in the lymph nodes, bones, and lungs. The results of the present preliminary trial warrant a further clinical trial in a larger patient population and using more reliable reference standards for the diagnosis of metastatic lesions to determine the usefulness of anti-^18^F-FACBC for staging and restaging of prostate cancer.
